# Decoding the paradox: the impact of TMT cognition heterogeneity on the speed of strategic change

**DOI:** 10.3389/fpsyg.2026.1782852

**Published:** 2026-04-10

**Authors:** Xiaohan Wang, Wei Miao

**Affiliations:** Veritas Collegiate Academy, Chesapeake, VA, United States

**Keywords:** cognition heterogeneity, relationship conflict, speed of strategic change, task conflict, top management team

## Abstract

**Objective:**

Although scholars have substantially explored the impact of top management team (TMT) on strategic change, how TMT composition influences the speed of strategic change is still under investigation. We move beyond TMT heterogeneity in demographic attributes and investigate the impact of TMT cognition heterogeneity on the speed of strategic change, considering the moderating role of TMT conflict.

**Method:**

Data was collected through a questionnaire survey. We randomly selected our sample firms from six different regions in eastern, central and western China. The final sample size is 132 Chinese firms. Due to the categorical nature of our data, the OSR (Optimal Scaling Regression) model was adopted to examine proposed hypotheses.

**Results:**

Our findings suggest that during the process of making strategic change, because positive influences are dominant when heterogeneity level is low while negative influences become dominant when heterogeneity level turns to high, TMT cognition heterogeneity drives up the speed of strategic change but at a decreasing rate. Moreover, the two types of TMT conflict moderate the impact of TMT cognition heterogeneity on the strategic change speed in opposite ways; that is, task conflict strengthens the relationship while relational conflict weakens it.

**Conclusion:**

First, our examination decodes the seemingly parodoxical impact of TMT cognition heterogeneity and thus enriches the research on the impact of TMT composition on strategic change. Second, we distinguish task conflict from relationship conflict and findings suggest that TMT task conflict promotes the impact of TMT cognition heterogeneity while TMT relationship conflict always hurts. Third, investigating the interaction effects of team processes (i.e., cognition diversity and team conflict) on strategic change, the complexity of information processing in decision making is articulated.

## 1 Introduction

Strategic change is fundamentally important to enterprises in dynamic environments, because enterprises need to overcome organizational inertia (Hannan and Freeman, 1984; Rumelt, 1995) and adapt to environmental changes (Kraatz and Zajac, 2001; MacKay and Chia, 2013; Rajagopalan and Spreitzer, 1997). Especially in current changing business environment due to geopolitics and digitalization (Mueller-Saegebrecht and Walter, 2025; Teece, 2025), exploring business opportunities quickly and making rapid strategic change by leveraging dynamic capabilities becomes critical for firms to gain and maintain competitive advantages over their competitors (Abernethy et al., 2021; Helfat et al., 2007; Yi et al., 2015). While top management team (TMT) is central to strategic decision making, during the process of making strategic change, strategic decisions may become challenging because of potential tension between team members and differences in TMT cognition when facing environmental complexity (Helfat and Peteraf, 2015).

Prior research has largely examined how TMT attributes, especially demographic characteristics and personality, affect organizational outcomes, such as strategic change (Bromiley and Rau, 2016; Carpenter et al., 2004; Herrmann and Nadkarni, 2014). However, how TMT composition influence the process of strategic change has not been investigated sufficiently, especially from the perspective of TMT cognition (Helfat and Peteraf, 2015; Wilms et al., 2019). TMT cognition, as a key dimension of dynamic managerial capabilities (Baishya et al., 2025), assumes a central role in initiating strategic change (Helfat and Martin, 2015). Heterogeneity of TMT cognition reflects diversity in TMT mental activities performed (Bhansing et al., 2012). Because of its possible positive effects to create more solution alternatives and negative effects to deter effective team coordination may coexist when influencing strategy process, a paradoxcial situation may happen. Moreover, a crucial drawback of heterogeneous teams is conflict (Greening and Johnson, 1997), leading to a lack of mutual attraction and interpersonal connection (Ndofor et al., 2015). Team conflict is thus always challenging, because the tension between team members can hinder team effectiveness (De Dreu and Weingart, 2003a), especially when firms make decisions on strategic changes when adapting to turbulent environment. To understand the effect of TMT cognition heterogeneity on strategic change speed, our research questions ask: *what is the relationship between TMT cognition heterogeneity and the speed of strategic change, and how the relationship is moderated by TMT conflict?*

The speed of strategic change refers to the time that firms take to change strategies, including the decision-making speed (Barm and Wally, 2003; Eisenhardt, 1989) and the implementation speed (Dooley et al., 2000). Strategic decision-making speed reflects the time it would take to initiate and formulate a strategic decision (Barm and Wally, 2003). And implementation speed is defined as “the time between when the commitment to act on the decision was made, to the time the decision was fully integrated into your organization’s operations” (Dooley et al., 2000, p. 1,245). As such, we define the speed of strategic change as the time spent on the process of formulating and implementing strategic change (Dooley et al., 2000; Eisenhardt, 1989). TMT cognition refers to mental activities that are performed with managerial cognitive capabilities (Helfat and Peteraf, 2015; Jin et al., 2026) and we propose that with TMT cognition heterogeneity, executives perform divergent mental activities that are able to influence the process of strategic change. Drawing from the information processing perspective, when diversity in TMT cognition is relatively low, it helps executives to engender more alternatives and thus promotes strategic change speed. But when TMT cognition heterogeneity increases to a high level, information processing will be deterred due to high team disagreement, resulting in a negative impact on strategic change speed. Thus, we argue that TMT cognition heterogeneity influences the speed of strategic change in a non-linear way; that is, TMT cognition heterogeneity drives the speed of strategic change until a threshold, after which the effect becomes negative.

Teams confront conflict when the tension between team members exists (De Dreu et al., 1999; Liu et al., 2022). Yu and Zellmerbruhn (2017) stated that team conflict is harmful to teams, because the potential benefits of conflict are difficult to be identified (Brattstrom, 2024; De Dreu and Weingart, 2003b; Todorova et al., 2022). However, prior research suggests that from an information-processing perspective, task conflict may be beneficial (de Wit et al., 2013; De Dreu and Weingart, 2003a) while relationship conflict is always detrimental (de Wit et al., 2012; Yang and Mossholder, 2004). We thus incorporated team conflict as a typical team process that may influence the impact of TMT cognition on strategic change speed, but the influence varies depending on the type of conflict. Specifically, we argue that TMT task conflict strengthens the impact of TMT cognition heterogeneity on the speed of strategic change while TMT relationship conflict weakens the relationship. Based on an analysis of survey data collected from 132 firms, our hypotheses have obtained substantial support.

This study contributes to established strategic change, team conflict, and TMT heterogeneity literatures in several ways. First, while managerial cognition functions as one core component of dynamic managerial capabilities (Adner and Helfat, 2003; Baishya et al., 2025), examining how managerial cognition influences the speed of strategic change yields novel empirical support for the significance of dynamic managerial capabilities in shaping strategic change within organizations (Helfat and Martin, 2015). By moving beyond heterogeneity in demographics and introducing a new dimension of heterogeneity, we examine the effect of TMT cognition heterogeneity on the speed of change. Our examination decodes the seemingly parodoxical impact of TMT cognition heterogeneity and thus enriches the research on the impact of TMT composition on strategic change. Second, we distinguish task conflict from relationship conflict by testing their respective moderating effects. Findings suggest that TMT task conflict promotes the impact of TMT cognition heterogeneity while TMT relationship conflict always hurts. Thus, we add new evidence to the team conflict research (de Wit et al., 2013). Third, investigating the interaction effects of team processes (i.e., cognition diversity and conflict) on strategic change, we aim to articulate the complexity of information processing in decision making. Findings suggest that the influence of TMT cognition heterogeneity on team outcomes varies, depending on the type of team conflict, which deepens the understanding of TMT heterogeneity from the information processing perspective.

## 2 Theoretical background and hypothesis development

### 2.1 Speed of strategic change

Strategic change is central to the adaptation to dynamic environment change, especially in the current VUCA era (Mueller-Saegebrecht and Walter, 2025; Teece, 2025; Wu et al., 2025). Rajagopalan and Spreitzer (1997) define it as a difference in the form, quality, or state over time of an organization’s alignment with its external environment. Recent advance in this stream of literature focuses on a continuous and evolving view of strategic change (Abernethy et al., 2021; Quigley and Hambrick, 2012; Schepker et al., 2017), especially in highly uncertain and volatile markets (Zhou et al., 2006). It mainly includes the magnitude (i.e., the change of strategic group) (Boeker, 1997; Zajac et al., 2000; Cho and Hambrick, 2006), and the speed (Adomako et al., 2021; Gersick, 1994; Yi et al., 2015), which refers to the length of time that a certain strategic change takes (Gersick, 1994).

Both magnitude and speed of strategic change are of essential importance to firms that compete in dynamic environments. Nevertheless, prior research has largely focused on the magnitude of change (e.g., Cho and Hambrick, 2006; Müller and Kunisch, 2018; Wu et al., 2011), neglecting the influence of the change speed. Yet, implementing strategic change at a high speed may allow a firm to seize more opportunities than its competitors, enabling it to gain an advantage (Lieberman and Montgomery, 1998).

Previous research has mostly investigated the impact of senior executives’ individual attributes on strategic change, such as personality (Herrmann and Nadkarni, 2014) and demographic characteristics (Triana et al., 2019; Wu et al., 2021). Despite the well-acknowledged importance of senior executives’ cognitive influence on strategy process (Bromiley and Rau, 2016), empirical examination has been scare. Therefore, in this study we focus on strategic change speed and investigate the impact of TMT cognition heterogeneity.

### 2.2 TMT cognition heterogeneity

The TMT cognition has been acknowledged to affect the microfoundations of dynamic capabilities (Duran and Aguado, 2022; Teece, 2007). In strategic management research, TMT cognition as well as its heterogeneity can be analyzed by information structures (Gary and Wood, 2011; Jin et al., 2026). Since the utilization and change in information structures are perceived as mental activities, TMT cognition influences executives’ capability to conduct mental activities when “acquiring and processing information” (Colman, 2006) and thus affects a firm’s strategic change (Helfat and Peteraf, 2015). There are two modes of mental activities: automatic mental activities that can respond quickly to external changes and controlled mental activities that can be relatively slow and respond more deliberately (Schneider and Shiffrin, 1977). Through controlled mental activities, senior managers cognitively process information to cope with non-routine events (Loring, 1999; VandenBos, 2007), such as strategic change.

Heterogeneity of TMT cognition refers to the level of diversity in TMT cognition (Jin et al., 2026) and influences the process and outcomes of strategic change (Helfat and Peteraf, 2015). It has been widely acknowledged to influence the process of strategic change, but the influence has not been adequately examined. Especially when investigating the role of cognition heterogeneity, empirical findings are inconclusive due to its possible positive and negative effects on team processes (Miller et al., 1998). On the one hand, diversity in TMT cognition may engender disagreement within the team, and thus encourage debate for more alternatives during the information processing (Bantel and Jackson, 1989; Wiersema and Bantel, 1992). On the other hand, disagreement between team members may make them less cohesive to each other (Aronson and Worchel, 1966; Condon and Crano, 1988) and hinder the effectiveness of communication within the team (Daft and Lengel, 1986). Hence, because the possible positive and negative effects of TMT cognition heterogeneity may exist at the same time when influencing strategic change speed, one typical dimension of strategy process, a paradoxcial situation may happen. We thus conclude that the situation may indicate a non-linear relationship between TMT cognition heterogeneity and the speed of strategic change, which is worthy of investigation.

### 2.3 Team conflict

Heterogeneous teams lack social closure and group identity and thus are more liable to generate conflict (Ndofor et al., 2015). Team conflict may engender tension within teams, and thus team members will be distracted when performing their tasks (Brattstrom, 2024; De Dreu and Weingart, 2003b). From an information-processing perspective, Jehn (1994, 1995, 1997) distinguish the types of conflict and note that task conflict can facilitate the team effectiveness on non-routine tasks while relationship conflict is always detrimental. For non-routine tasks that are commonly complex, teams do not have standardized solutions but need to search for alternatives. With the existence of task conflict, team members are liable to investigate emerged issues through delicate information processing of task-related information (Brattstrom, 2024; Jehn, 1995). This process encourages learning and creative thinking, resulting in more effective team outcomes (De Dreu and West, 2001). However, routine tasks have commonly been established well-developed and standard solutions. Hence, the existence of task conflict will interrupt the implementation of standard procedures rather than improving for solutions (Liu et al., 2022; De Dreu, 1997; De Dreu and Weingart, 2003a). In contrast, relationship conflict is always detrimental to team outcomes, because team members may need to cope with relational issues rather than concentrating on solving issues, resulting in dysfunctional team behaviors and subsequent lower information processing ability (de Wit et al., 2013; McShane and Von Glinow, 2000; Rollinson, 2002).

Previous research has investigated the two different conflict types and their impact on firm behaviors, respectively, (e.g., O’Neill et al., 2018; Puck and Pregernig, 2014; Todorova et al., 2022). However, how team conflict interacts with TMT composition to influence strategy process has been seldom examined. In this study, we investigate team conflict at the TMT level, because a top management team, as a team, plays a crucial role in firm strategic decision making (Hambrick and Mason, 1984). Following Jehn (1994, 1995, 1997), we differentiate the type of team conflict and examine different moderating effects of TMT task conflict vs. TMT relationship conflict. Focusing on the speed of strategic change, we investigate whether and how TMT task conflict may influence the baseline relationship differently from TMT relationship conflict.

### 2.4 TMT cognition heterogeneity and the speed of strategic change

The TMT cognition heterogeneity may have both positive and negative effects on TMT process outcomes. From an information-processing perspective, TMT cognition diversity may encourage debate and discussion, resulting in more alternatives for strategic decisions (Bantel and Jackson, 1989; Duran and Aguado, 2022), leading to greater team creativity (Bhansing et al., 2012) and planning openness (Bantel, 1994). In contrast, diversified cognition within the team may deter effective intra-group communication and collaboration (Daft and Lengel, 1986) and thus reduce team effectiveness (Helfat and Peteraf, 2015). In this study, we argue that TMT cognition heterogeneity influences the speed of strategic change in a non-linear way.

When TMT cognition heterogeneity is at a low-to-moderate level, diversified cognition within the team enables team members to possess a broader range of information sources and enlarge its knowledge base (Hambrick et al., 1996). In dynamic environment, heterogeneity in cognition may stimulate team members to debate on strategic decisions, which allows senior executives to realize more potential issues and acknowledge more perspectives to solve issues, and thus more alternative solutions to initiate change fast (Bantel and Jackson, 1989; Wiersema and Bantel, 1992). Moreover, with diversified cognition, team members are more open to new sources of information (Bantel, 1994). TMT members thus can seek and utilize diversified information sources (Ndofor et al., 2015), for example, to increase the speed of strategic change. Further, when TMT cognition heterogeneity is low, team members are mostly homogeneous in cognition and may have similar perspectives, preferences and skills. Thus, the possible tension between members will be limited. Hence, necessary coordination and communication to formulate and implement strategies will not be hindered and thus the speed of strategic change can be fast (Hambrick et al., 1996). As such, an overall positive effect of cognition heterogeneity on the speed of strategic change will emerge.

However, as the level of TMT cognition heterogeneity increases, cognition diversity within the team creates strong disagreements between team members (Danneels, 2011; Fredrickson and Mitchell, 1984). During information processing, such disagreements may lead to disputes rather than sharing ideas to engender more alternatives for fast strategic change (Glick et al., 1993). Moreover, strong intragroup disagreements imply the existence of apparently different values and views, which may deter necessary communication and coordination within the team (Miller et al., 1998), while effective collaboration and coordination is needed to complete the task (Ndofor et al., 2015). As such, they may not be able to evaluate possible resolutions and formulate and implement strategic change at a fast speed due to the communication and coordination failure. Therefore, we propose an inverted U-shaped relationship between TMT cognition heterogeneity and the speed of strategic change.

*Hypothesis 1: TMT cognition heterogeneity drives the speed of strategic change at a decreasing rate; that is, TMT cognition heterogeneity is positively related to the speed of strategic change at a low-to-moderate level and the impact turns to negative when TMT cognition heterogeneity becomes high.*

### 2.5 The moderating role of TMT conflict

TMT task conflict is task-related conflict and happens when tension emerges during resource allocation, procedure and policy distributions as well as information comprehension (de Wit et al., 2013; De Dreu and Weingart, 2003a; Simons and Peterson, 2000). From an information-processing perspective, we argue that low TMT task conflict may strengthen the positive effect of TMT cognition heterogeneity while high TMT task conflict is always detrimental to the impact of TMT cognition heterogeneity.

On the one hand, a low-to-moderate level of task conflict strengthens the positive effects of TMT cognition heterogeneity. First, taking strategic change is a non-routine task, which is complex without well-established solutions and thus requires within-group discussion (Jehn, 1995). Team members tend to discuss task-related issues and share ideas through processing of information for solutions. Consequently, creative thinking may be encouraged (De Dreu and West, 2001; Chung et al., 2015; Liu et al., 2022) to further promote the benefits of TMT cognition heterogeneity on how to take strategic change and thus facilitate the decision on strategic change. Second, task conflict between TMT members may help team members to more comprehensively understand the strategic issues and thus encourage team members to raise different types of solutions (de Wit et al., 2013; Simons and Peterson, 2000). A more comprehensive understanding of TMT tasks, in this study formulation and implementation of strategic change, can enable senior executives to better take advantage of cognition heterogeneity. Third, task conflict implies that TMT members have disagreements on key tasks and relevant resource allocation. With different views on those key issues, executives are more likely to evaluate other members’ strategic propositions for feasibility, which may help to engender more alternative solutions (Li and Li, 2009). This can complement the benefits of TMT cognition heterogeneity and thus strengthen its impact on the formulation and implementation of strategic change.

On the other hand, a high level of task conflict always exerts negative influences on the effects of TMT cognition heterogeneity. First, high task conflict within the TMT can create cognitive overload (e.g., Carnevale and Probst, 1998). Cognitively diversified teams encounter disagreements (Danneels, 2011). Cognitive overload due to high task conflict may exaggerate the disagreements within the team. Second, interpersonal conflicts may be exaggerated in teams with high task conflict (e.g., Greer et al., 2008; Simons and Peterson, 2000). As such, negative emotions may be triggered to reduce collaborative problem-solving and take time away from task-focused activities (de Wit et al., 2013; Todorova et al., 2014). Hence, a cognitively diversified team with exaggerated interpersonal conflicts further hurts team communication effectiveness, thus deterring the formulation and implementation of strategic change. Therefore, we propose that.

*Hypothesis 2: TMT task conflict steepens the relationship between TMT cognition heterogeneity and the speed of strategic change.*

The TMT relationship conflict can be identified when differences exist in individual values, attitudes and taste (De Dreu and de Vliert, 1997; De Dreu and Weingart, 2003a). We argue that when relationship conflict emerges in a top management team, it may negatively affect the relationship between TMT cognition and the speed of strategic change, because of its potential detrimental influences to team process (de Wit et al., 2013).

First, TMT relationship conflict may distract team members from concentrating on key issues that the team needs to solve. Thus, the efficiency of information processing may be lowered (Wang et al., 2019). Second, relationship conflict also negatively affects communication effectiveness between TMT members and hurt within-group trust. Thus, team members may not be willing to accept others’ suggestions (Sawyer et al., 2006; Liu et al., 2009). Third, due to the existence of relationship conflict, team members have to spend time and energy to cope with interrelationships rather than thinking for solutions. Subsequently, firm resources may not be allocated effectively to address strategic issues (Prasad and Junni, 2017).

As such, when TMT relationship conflict exists, the positive effect of TMT cognitive heterogeneity on the speed of strategic change is mitigated while its negative effect strengthened. Therefore, we propose that.

*Hypothesis 3: TMT relationship conflict flattens the relationship between TMT cognition heterogeneity and the speed of strategic change.*

## 3 Methodology

### 3.1 Data collection

Data for this study was collected through a survey conducted between December 2020 and April 2021. The relatively compressed timeline ensures the consistency of external environment among all interviewed TMT members. To reduce potential bias due to economic and cultural differences across different regions in China, we chose firms from six different regions in eastern, central and western China; that is, the Yangtze River region, Pearl River region, Bohai Sea region, Western region, Northeastern region and Middle region (Yi et al., 2015). We randomly selected our sample firms from a list of registered enterprises provided the Economy Commerce Committee, an administrative government agency established to coordinate the local economic development (Yi et al., 2018). With the support of local authorities, we obtained a list of more than 1,000 firms, and randomly chose 360 firms as the target firms for our sample. Table 1 demonstrates the descriptive characteristics of the sample firms.

**Table 1:** Descriptive statistics of sample firms.

Items	Categories	Quantity	Percentage
Firm age	<3	7	5.56%
	3–5	12	9.52%
	6–10	40	31.75%
	>10	67	53.17%
Ownership	State-owned	36	28.57%
	Private	56	44.44%
	Foreign	23	18.25%
	other	11	8.73%
Scale (number of employees)	<50	18	14.29%
	50–100	19	15.08%
	101–300	24	19.05%
	301–1,000	24	19.05%
	>1,000	41	32.54%
Development stage	Initial	12	9.52%
	Growth	53	42.06%
	Mature	48	38.10%
	Transformation	13	10.32%

We designed our original questionnaire in English based on the top management team and strategic change literatures following the three steps. First, translated the questionnaire was translated into Chinese by four bilingual experts. Second, a third party was involved to translate the Chinese version back into English and compared to the original English version to ensure consistency and accuracy (Brislin, 1970). Third, we conducted a pilot test with five managers. We checked each question with them to guarantee the accurate understanding of every question. Further, to ensure that each question can be understood correctly, detailed instructions were provided.

Our respondents were senior executives, identified to be TMT members, such as CEOs and other senior executives (e.g., CFO/COO/SVP), because senior executives commonly possessed accurate information and were knowledgeable about their firms’ strategic management practices. To enhance our data reliability, we required at least three TMT members of each firm to answer our questionnaires independently, which reduced the potential for generating artifactual bias (Podsakoff and Organ, 1986). Questionnaires were sent out to the randomly-selected 360 enterprises and totally 530 answers were collected from 178 firms, suggesting a 49% response rate. Potential non-response bias was tested between the 178 firms and the other firms that did not respond. We compared typical firm characteristics such as firm size, age and ownership status. Neither t-value between the two groups of firms was statistically significant. Hence, we concluded that the sample was representative.

Two criteria were adopted to drop off invalid answers of a questionnaire: (1) invalid if five continuous answers were not completed. (2) Invalid, if more than 10 continuous answers were the same. Following these criteria, a final sample of 335 validated responses from 132 firms were identified, which implies a 37% response rate, acceptable for research investigating top management teams (Baruch, 1999). The descriptive statistics of TMT members in our final sample firms is shown in Table 2.

**Table 2:** Descriptive statistics of top managers.

Items	Categories	Quantity	Percentage
Gender	Male	268	75.07%
	Female	89	24.93%
Age	26–30	33	9.24%
	31–35	79	22.13%
	36–40	85	23.81%
	41–45	89	24.93%
	>45	71	19.89%
Education level	Lower than college	25	7.00%
	College	48	13.45%
	Undergraduate	204	57.14%
	Masters	59	16.53%
	Ph. D.	21	5.88%
Tenure	<3	107	29.97%
	3–10	98	27.45%
	10–20	130	36.41%
	>20	22	6.16%

### 3.2 Measures

The dependent and independent variables were measured using the 5-point Likert scale. All items of each variable are shown in Table 3. We averaged the responses to each item for each firm used in the analyses.

**Table 3:** Factor loadings and coefficient alpha.

Factors	Items	Loading	Cronbach’s α
TMT cognition heterogeneity	How strongly do TMT members agree or disagree with each other about:		
	(1) What should the firm’s goal priorities be?	0.814	0.779 CR = 0.872 AVE = 0.694
	(2) The best way to ensure the firm’s long-run survival?	0.855	
	(3) Which organizational objectives should be considered the most important?	0.830	
TMT task conflict	(1) How much were disagreements over different ideas about business decisions?	0.822	0.732 CR = 0.849 AVE = 0.653
	(2) How much were differences about the content of the decisions that top managers have to work through?	0.842	
	(3) How much were differences of opinion within the team over decisions?	0.757	
TMT relationship conflict	(1) How much personal friction was there in your TMT during business decisions?	0.911	0.854 CR = 0.911 AVE = 0.774
	(2) How much were personality clashes between team members evident during decisions?	0.893	
	(3) How much tension was there in the TMT during decisions?	0.834	
Strategic change speed	(1) We design strategic plans very quickly.	0.753	0.870 CR = 0.914 AVE = 0.726
	(2) We implement strategic plans very quickly.	0.874	
	(3) Our top managers agree with each other rapidly on design and implementation of new strategies.	0.909	
	(4) Our employees accept firms’ new strategies or strategic adjustment very quickly	0.865	

The speed of strategic change (SSC). SSC represents the significant change in products/services and was measured using four items modified from the scales used by Dooley et al. (2000) and Kim and McIntosh (1996). As showed in Table 3, the items include: “relative to our major competitors, (1) We design strategic plans very quickly; (2) We implement strategic plans very quickly; (3) Our top managers agree with each other rapidly on design and implementation of new strategies; (4) Our employees accept firms’ new strategies or strategic adjustment very quickly” (Yi et al., 2015). Items were measured by using Likert scales ranging from 1 “no change” to 5 “strongly change.” Principal component analysis indicated that all items loaded on a single factor. The Cronbach’s coefficient alpha for this scale is 0.934 (see Table 3).

Cognition heterogeneity (CH). Following Miller et al. (1998) and Olson et al. (2007), we measured cognition heterogeneity by using three items, as showed in Table 3. Items were measured by using Likert scales ranging from 1 “strongly disagree” to 5 “strongly agree.” The Cronbach’s coefficient alpha for task conflict is 0.779.

The TMT task and relationship conflicts. Adopted from Li and Li (2009), TMT task conflict was measured using three items and relationship conflict was measured using three items represented the conflicts during the processes of decision-making, as shown in Table 3. Items were measured by using Likert scales ranging from 1 “no conflict” to 5 “lots of conflict”. The Cronbach’s coefficient alpha for task conflict is 0.732 and for relationship conflict is 0.854 (see Table 3).

Control variables. We controlled for a number of factors that might influence TMT characters (e.g., conflicts) and decision (e.g., speed of the firm’s strategic change). At the firm level, *Firm age* was measured by the number of years since the formation of the firm. Older firms often become more rigid and less able to change; they are subject to potential inertia as they age (Hannan and Freeman, 1984). *Firm size* is measured by the number of employees in a firm. Compared to large firms, smaller ones may own a relatively simple organizational structure and operational procedures. Type of firm ownership also is an important organizational characteristic that influences firm behavior, especially in the research context of China (Yi et al., 2018). Thus, we control *ownership structure* by asking respondents to indicate firms’ current ownership attribute: state-owned, privately owned, foreign (included JVs and wholly foreign-owned), domestic JVs, or others. *Organizational slack* has significant effect on firms to enable strategic changes (Yi et al., 2015). It is measured by asking respondents to indicate “whether focal firms can provide enough financial resources to business activities”. *Production capacity* also implies a firm’s capacity in initiating strategic changes (Yi et al., 2015) and thus was measured by asking respondents to indicate “whether focal firms own sufficient product capacity to business activities”.

At the TMT level, since the greater diversity of age among team members, the more conflict in the decision-making process (Harrison and Klein, 2007; Yi et al., 2018), we also controlled for *age diversity*, measured by standard deviation divided by the mean value (Ndofor et al., 2015), and *diversity of functional background*, measured with Blau’s (1977) Herfindal–Hirschman index, calculated as 1 − ΣSi2, where Si is the proportion of a TMT in the ith category (Wiersema and Bantel, 1992).

Further, to control for industry effects, we used a dummy variable to indicate *High-tech* firms; that is, firms located in high-tech industries were rated as 1 and the other firms were rated as 0.

### 3.3 Assessment of common method variance

Common method variance is mainly caused by the collection of dependent and independent variables in one survey from a single informant (Podsakoff et al., 2003). In order to minimize this potential threat, we split the surveys into three parts and invited three TMT members (i.e., CEO, CFO, COO etc.) from each firm to answer one part of the questionnaire, respectively. Following Podsakoff and Organ (1986), we also performed Harman’s one-factor test and a confirmatory factor analysis (CFA). If common method variance exerts serious threat for this study, a single factor accounting for most of the covariance may emerge. Our results show that four factors emerged in the unrotated factor structure, with the largest factor accounting for only 21.09% of the total variances. The CFA results suggest that the model assuming a single factor did not fit the data well (Chi-square/df = 9.28, RMSEA = 0.26, CFI = 0.55, NFI = 0.52, IFI = 0.55, and GFI = 0.57). After removing the common factor from the model and allowing all items to be assigned to their theoretical factors, the model fits the data well (Chi-square/df = 1.70, RMSEA = 0.08, CFI = 0.95, NFI = 0.91, IFI = 0.95, and GFI = 0.90). As such, we conclude that common-method bias is not a serious concern in the study (Sabherwal and Becerra-Fernandez, 2005).

### 3.4 Reliability and validity

We evaluated reliability and validity of all the measures. As shown in Table 3, the composite reliabilities were all above 0.60 and the factor loadings of all constructs were significantly above 0.60, suggesting sufficient convergent validity (Bagozzi and Yi, 1988). We also examined whether the average variance extracted (AVE) estimates for individual constructs were higher than the shared variances between the pairs of constructs to assess discriminant validity (Fornell and Larcker, 1981). As shown in Table 4, the square roots of the AVE for each construct along the diagonal (the bold figures) were largely higher than the correlations (Fornell and Larcker, 1981), suggesting adequate discriminant validity.

**Table 4:** Descriptive statistics bivariate correlations.

Variables	1	2	3	4	5	6	7	8	9	10	11	12
1. Firm age	–											
2. Firm size	0.447^**^	–										
3. Ownership structure	0.243^**^	−0.129	–									
4. High-tech	0.062	0.131	0.226^*^	–								
5. Age diversity	0.055	0.170	−0.034	0.027	–							
6. Diversity of functional background	−0.120	−0.013	−0.003	0.090	0.182^*^	–						
7. Organizational slack	−0.102	−0.056	−0.046	−0.113	−0.103	0.001	–					
8. Production capacity	0.033	0.164	0.084	0.021	0.084	−0.217^*^	0.348^**^	–				
9. TMT Cognition heterogeneity	0.077	−0.045	0.023	−0.062	−0.031	−0.015	0.359^**^	0.214^*^	(0.833)			
10. TMT Task conflict	0.105	0.169	0.012	0.306^**^	−0.051	−0.097	−0.145	−0.094	−0.195^*^	(0.803)		
11. TMT Relation conflict	−0.098	−0.038	0.157	0.158	0.045	−0.003	−0.124	0.061	−0.295^**^	0.472^**^	(0.880)	
12. Speed of strategic change	−0.031	−0.028	−0.002	0.091	0.012	0.061	0.375^**^	0.269^**^	0.393^**^	−0.011	−0.139	(0.852)
Mean	2.532	3.405	2.048	1.191	0.528	0.545	3.301	3.167	4.000	2.825	2.336	2.532
SD.	1.149	1.438	0.884	0.394	0.144	0.185	0.940	0.970	0.656	0.763	0.805	0.830

*N* = 132; *p < 0.05, **p < 0.01.

## 4 Results

In Table 4, we reported the means, standard deviations, and correlations between the dependent, independent, and control variables. In our analysis, the highest variance inflation factor (VIF) is 3.28, indicating that the potential multicollinearity issues do not exist.

Due to the categorical nature of our data, we adopted the OSR (Optimal Scaling Regression) model to examine our proposed hypotheses, which has been well acknowledged in prior management studies (e.g., Li et al., 2010). Categorical data may lead to an uneven distribution, which cannot be well addressed by the ordinary least square (OLS) regression model (Didow et al., 1985). The application of the OSR model allows us to use a regression model that can better address the condition of an uneven distribution through transforming the original variables (Ma et al., 2014). We further conducted hypothesis testing by OLS models and obtained results with weaker significance, indicating that in contrast to an OLS model, an OSR model fits our data better.

The independent variables and moderators are standardized because the need to generate the second- and third-tier interaction terms (Cohen and Cohen, 1983). The regression results of our hypotheses are shown on Table 5. All indicated significance levels are with a two-tailed test.

**Table 5:** Regression results.

Variables	The Speed of Strategic Change (SSC)			
	Model 1	Model 2	Model 3	Model 4
Controls				
Firm age	0.034 (0.086)	0.026 (0.085)	0.054 (0.083)	0.030 (0.082)
Firm size	−0.035 (0.090)	−0.124 (0.090)	−0.173^+^ (0.090)	−0.181^*^ (0.089)
Ownership structure	−0.200^*^ (0.083)	−0.165^*^ (0.082)	−0.139^+^ (0.081)	−0.170^*^ (0.080)
High-tech	0.141^+^ (0.080)	0.132 (0.083)	0.120 (0.081)	0.156^+^ (0.081)
Age diversity	0.040 (0.082)	0.030 (0.081)	0.056 (0.077)	0.026 (0.077)
Diversity of functional background	0.058 (0.081)	0.095 (0.080)	0.074 (0.076)	0.071 (0.076)
Organizational slack	0.415^***^ (0.087)	0.322^***^ (0.094)	0.395^***^ (0.093)	0.370^***^ (0.101)
Production capacity	0.160^*^ (0.083)	0.174^**^ (0.083)	0.096 (0.078)	0.128^+^ (0.077)
Main effects				
TMT Cognition heterogeneity (CH)	0.182^*^ (0.083)	0.198^**^ (0.089)	0.236^***^ (0.087)	0.228^***^ (0.087)
TMT Cognition heterogeneity square (CH^2^)	−0.202* (0.082)	−0.190* (0.082)	−0.199 * (0.095)	−0.204^*^ (0.097)
TMT Task Conflict (TC)		0.268^***^ (0.096)	0.250^***^ (0.089)	0.318^***^ (0.101)
TMT Relationship Conflict (RC)		−0.246^***^ (0.088)	−0.399^***^ (0.099)	−0.468^***^ (0.103)
Interactive effects				
CH * TC		−0.183^**^ (0.080)		−0.166^*^ (0.093)
CH^2^ * TC		−0.192^*^ (0.084)		−0.168^+^ (0.100)
CH * RC			−0.305^***^ (0.084)	−0.224^**^ (0.088)
CH^2^ * RC			0.389^***^ (0.098)	0.437^***^ (0.107)
*F*-value	2.578^**^	3.235^***^	3.278^***^	3.125^***^
*R* square	0.249	0.474	0.522	0.551
Adjusted *R* square	0.152	0.327	0.363	0.375
Max VIF	1.468	2.304	2.165	3.328

*N* = 132; ^+^*p* < 0.1, *p < 0.05, **p < 0.01, ***p < 0.001; Standard errors are in parentheses.

Hypothesis 1 argued that TMT cognition heterogeneity has an inverted U-shaped relationship with the speed of strategic change. Following the three-step approach suggested by Haans et al. (2016), we tested this effect. First, as shown in model 1 (Table 5), the coefficient of cognition heterogeneity is positive and statistically significant (0.182, *p* = 0.028), and the coefficient of cognition heterogeneity squared term is also negative and statistically significant (−0.202, *p* = 0.014). Second, the slopes of TMT cognition heterogeneity were tested. We found that when TMT cognition heterogeneity equals −3, 5 (minimum), the slope is 1.596 (*p* = 0.008) and when TMT cognition heterogeneity equals 1.5 (maximum), the slope is −0.424 (*p* = 0.073) thus, both slopes are steep sufficiently. Third, when TMT cognition heterogeneity is relatively low, it promotes the speed of strategic change. However, when it increases to a turning point (TMT cognition heterogeneity = 0.450, well within the data range of −3.5 to 1.5), the relationship turns to negative. Thus, hypothesis 1 is supported.

**Figure 1:**
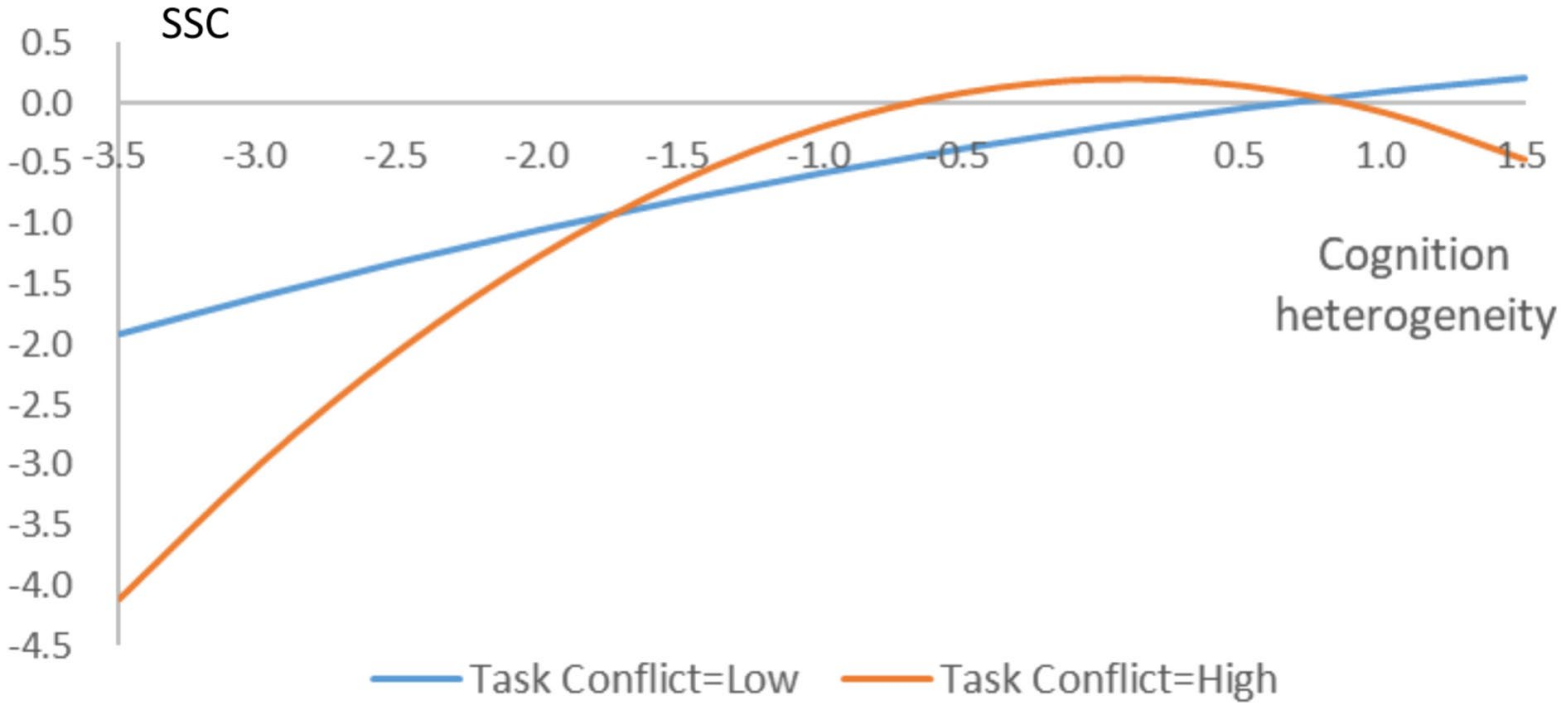
The moderating role of TMT task conflict. SSC, Speed of strategic change.

Hypothesis 2 proposed that TMT task conflict steepens the relationship between TMT cognition heterogeneity and the speed of strategic change. From model 2 in Table 5, we can see that the interactive coefficient between TMT cognition heterogeneity squared and TMT task conflict is negative and significant (−0.192, *p* = 0.022). For inverted U-shaped relationships, a steepening occurs when the coefficient of the interaction term is statistically negative (Haans et al., 2016, p. 1,187). Thus, hypothesis 2 receives support. The interaction effect is plotted at one standard deviation (SD) above or below the mean (Cohen and Cohen, 1983). As shown in Figure 1, when TMT task conflict is low, the turning point moves upward and to the right (TMT cognition heterogeneity = 7.659); that is, within the data range, the relationship is largely positive. However, when TMT task conflict is high, the turning point moves downward and to the left (TMT cognition heterogeneity = 0.009). These pieces of evidence provide support for a steepened inverted U-shape.

**Figure 2:**
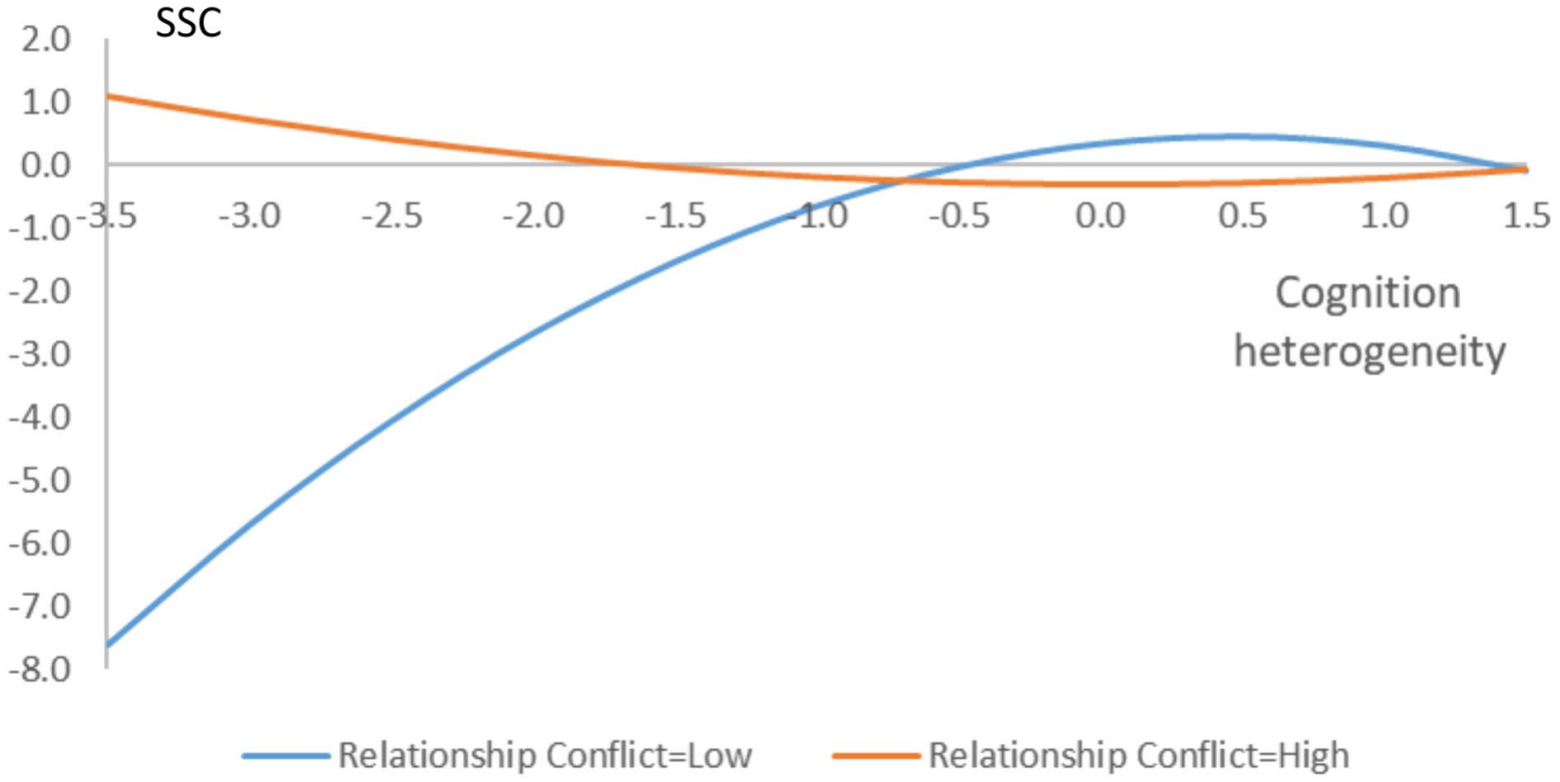
The moderating role of TMT relationship conflict. SSC, speed of strategic change.

Hypothesis 3 proposed that TMT relationship conflict negatively moderates the relationship between TMT cognition heterogeneity and the speed of strategic change. From model 5 in Table 5, we can see that the interactive coefficient between TMT task conflict and cognition heterogeneity squared is positive and significant (0.389, *p* = 0.000). For inverted U-shaped relationships, a flattening occurs when the coefficient of the interaction term is statistically positive (Haans et al., 2016, p. 1187). Thus, hypothesis 3 receives support. The interaction plot is also plotted in Figure 2 at one standard deviation (SD) above or below the mean. As shown in the interaction plot in Figure 2, when TMT relationship conflict is high, the turning point moves downward and to the left (TMT cognition heterogeneity = 0.009) and the coefficient of square term turns to positive, suggesting a largely negative relationship. Nevertheless, when TMT relationship conflict is low, the turning point moves downward and to the right slightly (TMT cognition heterogeneity = 0.471), these pieces of evidence provide support for a flattened inverted U-shape.

## 5 Discussion

### 5.1 Research findings and contributions

With technological dynamics and increasingly intensive competition in the VUCA era, to speed up operations has become one of fundamental requirements for firms intending to survive and develop (Eisenhardt and Schoonhoven, 1990; Wu et al., 2025). Filling in the gap in terms of the influences of TMT composition attributes on strategic change, this study focuses on the role of TMT cognition heterogeneity in the speed of strategic change. We also incorporate team conflict into our contingency investigation. Our findings suggest an inverted-U relationship between TMT cognition heterogeneity and the speed of strategic change; that is, TMT cognition diversity positively influences strategic change speed but at a decreasing rate. Moreover, TMT task conflict and relationship conflict moderate the relationship in different ways. Our findings provide support to our arguments that while TMT task conflict is beneficial when at a low level and its moderating impact turns to negative when it is at a high level. Nevertheless, the moderating effect of TMT relationship conflict is always detrimental.

Findings of this study thus enrich research on strategic change, team conflict, and TMT heterogeneity in several ways. First, we move beyond TMT demographic heterogeneity and tested the effect of TMT cognition heterogeneity on the speed of strategic change from an information processing perspective. Our investigation answers the call for more research on TMT cognitive role in strategy process (Bromiley and Rau, 2016; Müller and Kunisch, 2018). Our findings support an inverted U-shaped relationship, which suggests that a low level of TMT diversity in cognition promotes the speed of strategic change, while highly diversified TMT cognition hinders the speed. This evidence explains that when positive and negative effects coexist, how TMT cognition heterogeneity plays its role in strategy process and future research may examine whether an optimal level exists to influence team outcomes. Thus, by introducing a new dimension of heterogeneity to TMT composition, the investigation of this relationship contributes not only to the TMT composition literature but also to the strategic change literature.

Second, this study contributes to the research on team conflict by examining the moderating effect of TMT conflict. Prior research states that positive effects of team conflict are “largely elusive” (Yu and Zellmerbruhn, 2017). Moreover, scholars on task conflict provide inclusive findings, either positive, negative, or insignificant (Liu et al., 2022; Puck and Pregernig, 2014; Todorova et al., 2022). Our findings suggest that the moderating role of task conflict is positive when the conflict level is relatively low and relationship conflict always hurts. Providing a possible answer to the inclusive findings on the effect of team conflict, this study enriches the team conflict research by proposing and verifying that the influence of team conflict on team outcomes not only depends on the type of team conflict but also on the team process attributes.

Third, examination of the interaction effects of team processes (i.e., team conflict and team cognition diversity) on strategic change also extends research on the TMT heterogeneity research. Emphasizing the complexity of information processing in decision making (O’Neill et al., 2018), this study finds that the impact of TMT cognition heterogeneity on strategic change speed varies by the type of team conflict, which deepens our understanding of TMT heterogeneity from the information processing perspective.

### 5.2 Managerial implications

Findings of this study provide rich implications to senior managers, especially those from countries with turbulent business environment. On the one hand, senior managers need to be aware that the managerial cognitive capabilities differ within top management teams and thus TMT cognition can be widely diversified. TMT cognition heterogeneity may both have possible positive and negative effects on team outcomes (Miller et al., 1998). When interacting with other team processes (team task conflict versus team relationship conflict in this study) on process outcomes (strategic change speed in this study), TMT cognition diversity may engender divergent effects. In another word, TMT cognition diversity may promote the speed of strategic change when the diversity level is low but is detrimental to strategic change speed when reaching a high level. Thus, top managers should acknowledge the level of TMT cognition heterogeneity within the team and take advantage of and TMT cognition heterogeneity. As such, they may take measures to maintain the level of cognition diversity within the team to a moderate level, since cognition diversity can be beneficial when the diversity level is moderate.

On the other hand, senior managers need to be aware that team conflict is always possible to influence the effect of TMT cognitive diversity in the speed of formulating and implementing strategic change. Meanwhile, senior executives should also acknowledge the possible different effects of task versus relationship conflict on the relationship. Realizing this fact can help senior managers to take effective measures to mitigate the negative influence of relationship conflict while taking advantage of low task conflict, if they aim to speed up firms’ strategic change.

### 5.3 Limitations and future research

This study has some limitations and thus leaves space for future research. First, to test our proposed hypotheses, we collected data from a sample of Chinese firms. China is an ideal research context when examining research questions in strategic change, because firms operating in China are experiencing turbulent environment (Yi et al., 2015). Chinese market is well known for its dynamic environment due to dramatic changes in factor and product markets as well as in policies, especially after China’s entry into the WTO in 2001 (Richard et al., 2019). However, the focus on Chinese sample firms may constrain the generalizability of our findings. Hence, future research may replicate the testing of our hypotheses in other research contexts with different levels of dynamic environment.

Second, this study focuses on TMT cognition and examines its impact on strategic change speed, given that managerial cognition is one core dimension of dynamic managerial capabilities that influences strategic change (Adner and Helfat, 2003; Helfat et al., 2007). Nevertheless, entering the 21st century, emerging geopolitics engenders great environmental dynamism (Baishya et al., 2025; Teece, 2025), which calls for more research to enrich the understanding of dynamic managerial capabilities’ impact on strategic change, such as the impact of managerial political capital (Baishya et al., 2025).

Third, this study investigates the relationship between TMT cognition heterogeneity and strategic change, focusing on the change speed. However, strategic change magnitude is another important dimension to comprehensively understand strategic change (Zajac et al., 2000; Cho and Hambrick, 2006). Future research may thus explore the relationship between a specific dimension of dynamic managerial capabilities and the magnitude of strategic change that has been under-investigated.

Fourth, although we have examined the interaction effect of specific TMT process variables (i.e., cognition heterogeneity and team conflict) on strategic change speed, other TMT processes may also influence the formulation and implementation of strategic change, such as TMT behavioral integration (Yi et al., 2018) and TMT cohesion (Vissa and Chacar, 2009). Hence, drawing from alternative theoretical perspectives, future research may incorporate other types of team processes and examine their specific interaction effect on strategic change.

Lastly, in our regression analysis, we included industry-, firm-, and TMT- levels of control variables. While incorporating multiple levels of controls enhances the rigor of our analysis, we acknowledge that there are more potential control variables, such as TMT experience diversity and international experience, that may exert influences on the speed of strategic change. Thus, future research may consider involving varying potential control variables in the survey design and demonstrate their effects through data analysis.

## 6 Conclusion

In the current VUCA era, ever changing environment requires firms’ senior executives to respond quickly by initiating strategic change quickly. While the importance of TMT composition on firms’ strategic change has been widely acknowledged, prior research mostly focuses on TMT demographic attributes. Moving beyond, this study investigates the impact of TMT cognition heterogeneity on the speed of strategic change. We argue that low cognition heterogeneity leads to a dominant positive influence while a high level influences detrimentally, resulting in a non-linear impact on the strategic change speed. Team conflict is further examined as a contingent team process factor, distinguishing the opposite moderating roles of task conflict vs. relationship conflict. In conclusion, this study not only decodes the paradoxical effects of TMT cognition heterogeneity, but also enhances our understanding of the interaction of team process on the speed of strategic change.

## Data Availability

The raw data supporting the conclusions of this article will be made available by the authors, without undue reservation.
